# Insecticide Resistance and the Future of Malaria Control in Zambia

**DOI:** 10.1371/journal.pone.0024336

**Published:** 2011-09-06

**Authors:** Emmanuel Chanda, Janet Hemingway, Immo Kleinschmidt, Andrea M. Rehman, Varsha Ramdeen, Faustina N. Phiri, Sarel Coetzer, David Mthembu, Cecilia J. Shinondo, Elizabeth Chizema-Kawesha, Mulakwa Kamuliwo, Victor Mukonka, Kumar S. Baboo, Michael Coleman

**Affiliations:** 1 Ministry of Health, National Malaria Control Centre, Lusaka, Zambia; 2 Liverpool School of Tropical Medicine, Liverpool, United Kingdom; 3 MRC Tropical Epidemiology Group, London School of Hygiene and Tropical Medicine, London, United Kingdom; 4 Malaria Research Programme, Medical Research Council, Durban, South Africa; 5 School of Medicine, University of Zambia, Lusaka, Zambia; 6 Ministry of Health, Headquarters, Lusaka, Zambia; London School of Hygiene and Tropical Medicine, United Kingdom

## Abstract

**Background:**

In line with the Global trend to improve malaria control efforts a major campaign of insecticide treated net distribution was initiated in 1999 and indoor residual spraying with DDT or pyrethroids was reintroduced in 2000 in Zambia. In 2006, these efforts were strengthened by the President's Malaria Initiative. This manuscript reports on the monitoring and evaluation of these activities and the potential impact of emerging insecticide resistance on disease transmission.

**Methods:**

Mosquitoes were captured daily through a series of 108 window exit traps located at 18 sentinel sites. Specimens were identified to species and analyzed for sporozoites. Adult *Anopheles* mosquitoes were collected resting indoors and larva collected in breeding sites were reared to F1 and F0 generations in the lab and tested for insecticide resistance following the standard WHO susceptibility assay protocol. Annual cross sectional household parasite surveys were carried out to monitor the impact of the control programme on prevalence of *Plasmodium falciparum* in children aged 1 to 14 years.

**Results:**

A total of 619 *Anopheles gambiae s.l.* and 228 *Anopheles funestus s.l.* were captured from window exit traps throughout the period, of which 203 were *An. gambiae* malaria vectors and 14 *An. funestus s.s.*. In 2010 resistance to DDT and the pyrethroids deltamethrin, lambda-cyhalothrin and permethrin was detected in both *An. gambiae s.s.* and *An. funestus s.s.*. No sporozoites were detected in either species. Prevalence of *P. falciparum* in the sentinel sites remained below 10% throughout the study period.

**Conclusion:**

Both *An. gambiae s.s.* and *An. funestus s.s.* were controlled effectively with the ITN and IRS programme in Zambia, maintaining a reduced disease transmission and burden. However, the discovery of DDT and pyrethroid resistance in the country threatens the sustainability of the vector control programme.

## Introduction

Malaria vector control activities are substantially increasing in many malaria endemic countries [1], with some countries considering elimination [2]. Indoor Residual Spraying (IRS) and Insecticide Treated Nets (ITNs) form the backbone of these activities and both have been proven as excellent vector control strategies [3]. However, the number of insecticides recommended for both methods is severely restricted, hence monitoring and management of insecticide resistance within the control programme is essential if control is to be maintained [4].

Zambia first initiated IRS with DDT in the 1950s, at the same time malaria became a notifiable disease [5]. IRS coverage was reduced by 30% by 1973 due to economic constraints and environmental concerns about the use of DDT. The IRS programme stopped completely in the mid 1980s. With reduced vector control and the development of parasite resistance to anti-malarial drugs [6], malaria cases increased from 121.5 per 1000 in 1976 to 394 cases per 1000 in 2002[5].

In 2000, Konkola Copper Mines, a private company, reintroduced IRS with pyrethroids and DDT in two districts in Zambia [7] The success of these IRS programmes led the National Malaria Control Programme (NMCP) to again implement an IRS programme alongside the distribution of Long Lasting Insecticide Treated Nets (LLINs) [8]. As these malaria vector control interventions are being scaled up their impact has been monitored [9].

In areas of hyper and holoendemic malaria, vector control is critical in reducing human malaria transmission and the related morbidity and mortality. Evidence-based deployment and optimal assessment of vector control interventions is needed to formulate operational control policies. This requires the collation and assessment of a number of different data systems. To facilitate data handling the Zambian NMCP evaluated the Malaria Decision Support System (MDSS) developed by the Innovative Vector Control Consortium (IVCC) [10].

## Materials and Methods

### Ethics clearance

Ethical clearance for this study was obtained from the University of Zambia Biomedical Ethical Committee (Assurance No. FWA00000338, IRB00001131 of IOR G0000774 reference code 002-07-07). Written informed consent was obtained from all participants in this study.

### Study sites and interventions

**Figure 1:**
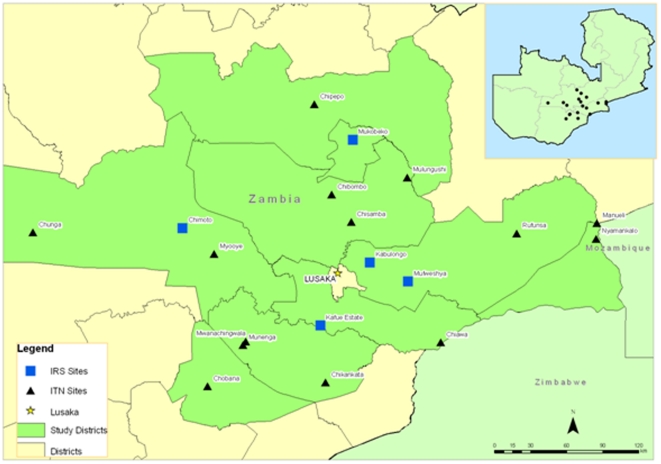
Map showing the spatial distribution of sentinel sites in Zambia.

Zambia is situated in the Southern African region with a population of approximately 13 million, 45% of whom are below the age of fifteen [11]. Data were collected from 18 sentinel sites, distributed amongst nine districts within a 350 km radius of the capital, Lusaka (Figure 1). Sites were selected to assist in monitoring the expanding vector control programme. At each site annual household surveys were carried out to measure *Plasmodium falciparum* prevalence in children aged 1 to 14. Relative mosquito abundance and infectivity were measured as a proxy for transmission and insecticide resistance status was monitored. Indoor residual spraying was carried out in October each year from 2003 to 2010 with DDT at 2 g/m^2^ (Avima, South Africa) in 5 sites only (Chimoto, Kabulongo, Kafue Estates, Mufweshya and Mukobeko). Perma Net®, (Verstargaard) and Olyset® (Sumitomo), LLINs were distributed to all areas (Figure 1). Countrywide mass distribution of ITNs commenced in 2005, prior to this ITN distribution was through the antenatal clinics and commercial outlets. Over 5 million ITNs, enough to cover 96% of Zambia population, had been distributed by 2008 [8,9]. All vector control was carried out by the NMCP as part of their ongoing operational control activities.

### *Plasmodium falciparum* prevalence surveys

Prevalence surveys were conducted at the end of the malaria transmission season in April/May in 2008, 2009 and 2010. Households were selected from strata formed by dividing sentinel sites into quadrants. In each quadrant a starting house was selected and all children aged 1 to 14 tested for *P. falciparum* infection using ICT™ malaria combo rapid diagnostic tests (R&R, Cape Town, South Africa). The investigator then proceeded to the next nearest house and tested all children until a total of 35 children per quadrant had been tested. Children testing positive for *P. falciparum* were offered treatment with Coartem® (artemether-lumefantrine) according to the National Malaria Control Programme (NMCP) guidelines. Any complicated malaria case was referred to the nearest health centre. The sentinel site specific sample size was calculated to provide evidence at the 5% significance level of an absolute reduction in *P. falciparum* prevalence of 20%. Prevalence of *P. falciparum* infection and 95% confidence intervals (CI) for each sentinel site were estimated taking account of clustering by sentinel site using the statistical software package STATA (StataCorp LP. Stata Statistical Software: Release 10. College Station, TX, USA.).

Sentinel sites were considered as the primary sampling unit. Logistic regression, allowing for complex survey designs, was performed to estimate the mean effect of the vector control intervention on prevalence compared to 2008 data.

### Mosquito Species Abundance and Infectivity

Window exit traps were fitted to six houses in each sentinel site with the homeowners consent in April 2008 and operated until May 2010. Houses were selected to cover the whole of a sentinel site. Each house was constructed in traditional style and in good repair with a window in the bedroom for the exit trap to be fitted. The Chunga sentinel site was not used for exit window trap collections, as houses were constructed from concrete blocks with corrugated metal roofs. Mosquito collections were made daily by homeowners trained to empty the contents of the window trap into pre-labelled specimen jars containing isopropanol. Checklists were completed specifying nights for which traps were not operating.

### Mosquito species identification

Anopheline mosquitoes were identified morphologically as *Anopheles gambiae* Giles complex, *An. funestus* Giles group or *An. nili* Theobald group [12,13]. Sibling species were identified and *An. gambiae s.s* were identified as M or S molecular form using PCR [14–18].

### Malaria transmission

DNA was extracted from the head and thorax of mosquitoes and tested for the presence of *P. falciparum* sporozoites [19]. Numbers of mosquitoes per trap per night were calculated for each vector species. Using the species specific estimated sporozoite prevalence the number of infectious mosquitoes per species per trap per night was calculated. The relative transmission index, defined as the ratio of infective numbers of mosquitoes per trap per night, relative to the baseline year of 2008, could not be computed because none of the specimens collected contained sporozoites [20].

### Insecticide Resistance

Mosquitoes were collected during the rainy seasons of 2009 and 2010 from 17 localities. *Anopheles* mosquitoes were collected between 06.00 and 08.00 either by larval collections from breeding sites, or as blood fed adult females resting inside homes. Collected mosquitoes were transported to the laboratory, and reared to adults or transferred to individual oviposition tubes and females allowed to lay eggs that were reared to adults at 26±2°C and 70–80% relative humidity.

Insecticide susceptibility assays were carried out on a random sample of 1-3 day old, sugar fed F1 and F0 adults following the procedure described by the World Health Organization (WHO 1998). Insecticides tested included bendiocarb (0.01%), DDT (4%), deltamethrin (0.05%), lambda-cyhalothrin (0.05%), malathion (5%), permethrin (0.75%), propoxur (0.1%).

Both Leu-Phe (west) and Leu-Ser (east) *kdr* mutations were investigated [21,22] using the taqman PCR [23].

## Results

### Malaria Prevalence in Children 1 to 14 years old

A total of 1,823 children aged between 1 to 14 years were tested for *P. falciparum* parasitaemia in all sentinel sites except Manueli and Nyamankalo in April/May 2008. Follow-up surveys were carried out in all sites in 2009 and 2010 with 2,176 children and 2,192 children respectively. The combined prevalence of infection with *P. falciparum* across all sites was 6.9% (95% CI  =  3.9–12.1) in 2008, 4.9% in 2009 (95% CI 1.7–13.4) and 6.8% in 2010 (CI = 2.2–18.9) (Table 1).

**Table 1:** Prevalence of infection with *Plasmodium falciparum* in children 1 to - 14 years of age, by sentinel site, observed during household surveys in 2008, 2009 and 2010 in Zambia.

	April/May 2008		April/May 2009		April/May 2010			
Sentinel site	PI (n)	95% CI	PI (n)	95% CI	PI (n)	95% CI	*P*(08-09)	*P*(09-10)
IRS Sites								
Chimoto	3.2 (93)	[0.8–12.8]	0.7 (141)	[0.1–5]	3.4 (145)	[1.1–9.9]	0.206	0.182
Kabulongo	11.4 (158)	[10.9–36.5]	0 (84)	-	4.6 (130)	[1.6–12]	**0.0007**	0.032*
Mukobeko	7 (157)	[3.3–14.1]	6.7 (134)	[3.1–13.8]	6.2 (130)	[3.2–11.4]	0.933	0.89
Kafue estate	2.3 (128)	[0.8–6.6]	0 (116)	-	3.7 (137)	[1.4–9.1]	0.129	**0.054**
Mufweshya	4.3 (69)	[1–17.1]	0 (73)	-	1.8(113)	[0.5–6.6]	**0.038**	0.18
ITN sites								
Chiawa	2 (148)	[0.5–8.1]	3.7 (134)	[1.4–9.5]	5.1 (136)	[2.3–11.2]	0.476	0.637
Chibombo	21.2 (146)	[12.7–33.3]	9.3 (161)	[4.5–18.2]	3 (132)	[1.3–7.1]	**0.0311**	0.072
Chikankata	1.1 (93)	[0.2–7.2]	0.7 (147)	[0.1–4.9]	0.7 (136)	[0.1–4.7]	0.765	1
Chipepo	11 (73)	[6.2–18.6]	4.1 (123)	[1.5–10.7]	5 (120)	[2.3–10.3]	0.076	0.766
Chisamba	0.9 (109)	[0.1–6.3]	0.7 (139)	[0.1–5.1]	2 (150)	[0.7–5.9]	0.874	0.429
Chobana	8.9 (79)	[3.5–20.5]	3.2 (124)	[0.7–13.2]	1 (97)	[0.1–7]	0.101	0.283
Chunga	3.6 (83)	[0.5–20.7]	4.2 (95)	[1.5–11.6]	1.9 (104)	[0.3–12.1]	0.83	0.352
Mwanachingwala	1.2 (86)	[0.2–7]	0 (152)	-	1.5 (131)	[0.2–10.6]	0.273	0.221
Mulungushi	15.2(46)	[7.8–27.5]	4.6 (131)	[1.5–13.2]	8.1 (123)	[3.8–16.5]	**0.0172**	0.326
Munenga	1.5 (134)	[0.4–5]	0 (138)	-	0 (134)	-	0.22	0
Myooye	0 (117)	-	0 (140)	-	3 (133)	[0.9–9.2]	-	0.083
Rufunsa	23.1 (104)	[11.6–40.6]	40.7 (135)	[30.1–52.4]	58.2 (141)	[46.5–69]	**0.0275**	0.078
All	6.9 (1823)	[3.9–12.1]	4.9 (2167)	[1.7–13.4]	6.8 (2192)	[2.2–18.9]	0.036	**0.031**
IRS	6.3 (605)	[3.4–11.5]	1.8 (548)	[0.4–8.4]	4.0 (655)	[2.9–5.5]	0.10	0.22
ITN	7.2 (1218)	[3.3–15.2]	5.9 (1619)	[1.9–17.3]	7.9 (1537)	[2.0–26.3]	0.60	0.11

PI  =  Prevalence of infection,% 95% CI  =  95% Confidence interval **Bold  = ** Change since 2008 was statistically significant.

No significant change in prevalence was observed in all ITN sites between years 2008, 7.2 (95% CI  =  3.3–15.2), 2009, 5.9 (CI 95%  =  1.9–17.3) to 2010, 7.9 (95% CI  =  2–26.3). In IRS sites there was a significant overall decline in prevalence of infection between 2008 and 2009 from 6.3 (95% CI  =  3.4–11.5) to 1.8 (95% CI  =  0.4–8.4) (OR  =  0..28, 95% CI  =  0.05–1.44, *P* = 0.036). However, prevalence increased again in 2010 to 4.0 (95% CI  =  2.47–5.47) (OR  =  2.22, 95% CI  =  0.57–8.65, *P* = 0.031) (Table 1).

### Mosquito Species Abundance, sporozoite rate and transmission

A total of 619 *An. gambiae s.l.* and 228 *An. funestus s.l.* were collected and morphologically identified from 108 window exit traps operating continuously from April 2008 to May 2010. Five hundred and twenty five *An. gambiae s.l.* were subsequently identified to species level. There were four *An. gambiae s.s,* 199 *An. arabiensis and* 322 *An. quadriannulatus.* Two hundred and four *An. funestus s.l* were identified to species, these were 14 *An. funestus s.s*, 98 *An. parensis*, 20 *An. rivulorum*, 18 *An. leesoni,* 16 *An. vaneedeni,* 14 were identified as the recently described *An. funestus-*like, 1 *An.nili* s.s and 23 unidentified. Culicine mosquitoes were recorded in the absence of Anopheles to demonstrate that the traps were being operated correctly.

At 217, the numbers of actual vectors, *An. gambiae s.s., An. arabiensis* and *An. funestus s.s.* trapped was extremely low. All of these were tested for sporozoites and all were negative. Due to zero sporozoites a transmission index could not be calculated (Table 2).

**Table 2:** Vector Abundance, Infectivity by period of time and intervention type.

	All sites		All ITN sites		All IRS sites	
Year	04/08-4/09	05/09-5/10	04/08-4/09	05/09-5/10	04/08-4/09	05/09-5/10
***An. gambiae s.l***						
No. caught	409	210	395	195	14	15
No. analyzed for species id	360	167	354	157	6	10
An. arabiensis propn (%)	24.0	32.9	23.8	34.4	28.6	13.3
* An. gambiae* s.s propn (%)	0.49	0.95	0.51	1.03	0.00	0.00
***An. gambiae s.s***						
No. Estimated	2	2	2	2	0	0
No per trap per 100 nights	0.03	0.03	0.03	0.03	0.00	0.00
No. Sporozoite positive	0(n = 2)	0(n = 2)	0(n = 2)	0(n = 2)	0(n = 0)	0(n = 0)
***An. arabiensis***						
No. Estimated	113	86	104	83	9	3
No per trap per 100 nights	1.59	1.21	1.46	1.17	0.13	0.04
No. Sporozoite positive	0(n = 125)	0(n = 98)	0(n = 104)	0(n = 92)	0(n = 9)	0(n = 6)
***An. funestus s.l***						
No. caught	105	123	94	113	11	10
No. analyzed for species id	99	105	91	95	8	10
No. *An. funestus* s.s	8	5	8	4	0	1
*An. funestus* s.s propn (%)	7.62	4.07	8.51	3.54	0.00	10.00
***An. funestus s.s***						
No. Estimated	8	6	8	5	0	1
No per trap per 100 nights	0.12	0.08	0.12	0.07	0.00	0.01
No. Sporozoite positive	0(n = 8)	0(n = 9)	0(n = 8)	0(n = 6)	0(n = 0)	0(n = 1)

### Insecticide susceptibility

A total of 1,742 *An. gambiae s.s.* and 796 *An. funestus s.s.,* from 17 localities, 11 of which were sentinel sites were assayed for insecticide susceptibility using the WHO protocol [24]. Prior to 2009, no resistance had been detected to any insecticide in *An. gambiae s.l*. or *An. funestus* in Zambia (Table 3 and Table 4). Between 2009 and 2010 resistance to pyrethroids (deltamethrin, lambda-cyhalothrin and permethrin) and DDT was detected in both *An. gambaie s.s.*and *An. funestus s.s*. No resistance was detected to the carbamate (bendiocarb) or the organophosphate (malathion) in either species. Limited numbers of mosquitoes were found at all sites, which correlates with the species abundance calculated from the window trap collections.

**Table 3:** WHO susceptibility test results on 1–3 dayold *An. gambiae s.s* of 17 localities in Zambia.

	1999											
Location	deltamethrin (0.05%)		permethrin (0.75%)		λ-cyhalothrin (0.05%)		DDT (4%)					
	n	%	n	%	n	%	n	%				
**ITN only sites**81												
Chibombo*		100	-	-	-	-	11	100				
Chingola*	15	100	-	-	-	-	5	100				
Chipulukusu*			46	100^b^			121	100^b^				
Livingstone#	17	100	-	-	13	100	32	100				
Lusaka#	7	100	-	-			6	100				
Mukobeko#	19	100^a^	-	-	11	100	9	100				
Mushili	-	-	-	-	-	-	73	100^b^				
Samfya#	8	100			5	100	7	100				

	2009-2010											
Location	deltamethrin (0.05%)		permethrin (0.75%)		λ-cyhalothrin (0.05%)		DDT (4%)		Malathion (5%)		Bendiocarb (0.01%)	
	n	%	n	%	n	%	n	%	n	%	n	%
**ITN only sites**												
Chiawa	3	100	-	-	-	-	-	-	-	-	-	-
Chipepo	43	41.8	-	-	-	-	-	-	-	-	-	-
Mwanachingwala	4	75	-	-	-	-	-	-	-	-	-	-
Myooye	74	93.2	-	-	-	-	73	69	-	-	-	-
Nanga Farms	8	100	-	-	-	-	5	100	-	-	-	-
Nyamankalo	11	90.9	4	100	6	83.3	8	100			10	100
**IRS sites**												
Chipulukusu*	96	13.5	19	61	-	-	428	43	-	-	-	-
Kafue	-	-	-	-	-	-	8	100	-	-	-	-
Kizingezinge	105	95.2	-	-	-	-	157	3.8	-	-	-	-
Mukobeko#	16	100	-	-	-	-	-	-	-	-	-	-
Mushili	60	41	31	55	-	-	100	11	47	100	-	-

%  = percentage mortality.

_a_ = p>0.1,

_b_ = p<0.001,

* =  1999 Unpublished baseline data collected by TDRC,

# =  1999 Unpublished data collected by NMCP.

**Table 4:** WHO susceptibility test results on 1-3-d-old *An. funestus s.s* of 17 localities in Zambia.

Location	1999									
	deltamethrin (0.05%)		λ-cyhalothrin (0.05%)		DDT (4%)		Propoxur (0.01%)		Malathion (5%)	
	n	%	n	%	n	%	n	%	n	%
**ITN only sites**										
Chibombo*	72	100^b^	-	-	-	-	19	100	-	-
Chingola*	-	-	-	-	-	-	3	100	16	100
Livingstone#	5	100	7	100	6	100	-	-	-	-
Mukobeko#	25	100^a^	15	100	25	100	-	-	-	-
Mushili*	4	100	-	-	-	-	-	-	4	100

	2009-2010									
Location	deltamethrin (0.05%)		permethrin (0.75%)		λ-cyhalothrin (0.05%)		DDT (4%)		Bendiocarb (0.01%)	
	n	%	n	%	n	%	n	%	n	%
**ITN only sites**										
Chibombo*	9	88.9	-	-	-	-	-	-	-	-
Chipepo	4	100	-	-	-	-	-	-	-	-
Manueli	11	72.7	-	-	-	-	-	-	-	-
Mulungushi	-	-	-	-	4	100	-	-	7	100
Mwanachingwala	22	81.8	-	-	-	-	-	-	-	-
Myooye	27	96.2	-	-	-	-	62	94	-	-
Nanga Farms	30	100	11	90.9	-	-	10	100	-	-
Nyamankalo	87	80.5	-	-	-	-	33	88	-	-
Rufunsa	66	66.6	-	-	-	-	-	-	-	-
**IRS sites**										
Kabulongo	15	80	-	-	-	-	14	100	-	-
Kafue	23	95.6	-	-	-	-	90	98	-	-
Katete	-	-	-	-	-	-	5	100	-	-
Mufweshya	18	100	-	-	-	-	21	100	-	-
Mukobeko#	26	96	-	-	-	-	-	-	-	-

%  = percentage mortality.

_a_ = p>0.1

_b_ = p<0.001,

* =  1999 Unpublished baseline data collected by TDRC,

# =  1999 Unpublished data collected by NMCP.

**Figure 2:**
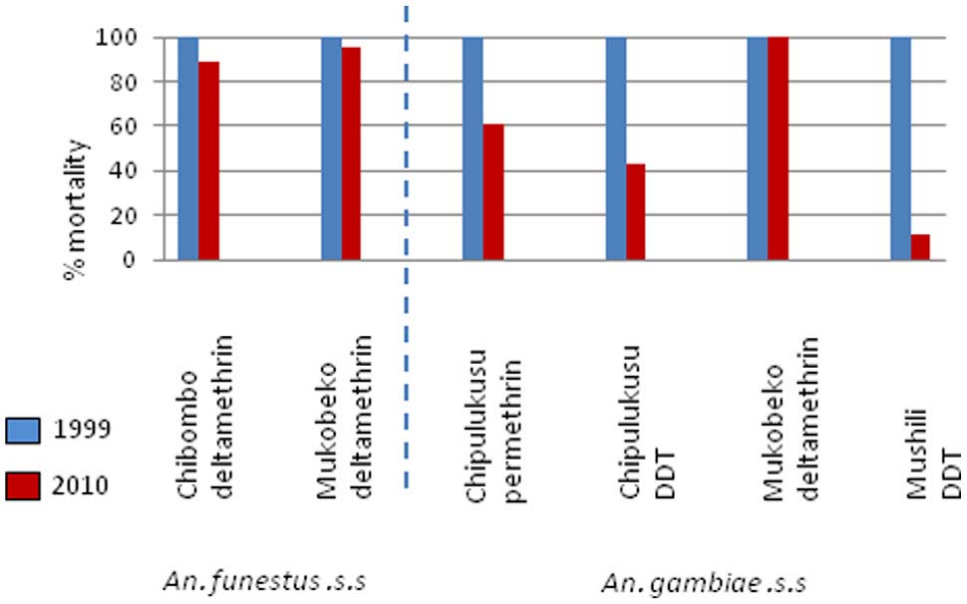
Comparison of insecticide resistance in *An. gambiae s.s.* and *An. funestus s.s.* from 1999 and 2010.

Prior to interventions mosquitoes from tested from 8 locations in Zambia were fully susceptible to the WHO discriminating dosage of deltamethrin, lambda-cyhalothrin, DDT, propoxur and malathion in 1999. Three of these locations, Chipulukusu, Mukobeko and Mushili had IRS introduced in 2003. In 2010, significant (P<0.001) resistance was detected in *An. gambiae s.s.*in Chipulukusu to deltamethrin (13.5%), permethrin (61%) and DDT (43%) and Mushili to DDT (55%). In Mukobeko no resistance was detected in *An. gambiae s.s.* to deltamethrin but a slight increase in resistance was detected in *An. funestus s.s.* (96% P<0.1). In Chibombo where only ITNs have been distributed resistance to deltamethrin was detected in *An. funestus s.s.* (88.9% P<0.001) in 2010 (Table 3 & 4 and Figure 2).

In 2010 new sites were surveyed for resistance. In the IRS sites *An. funestus s.s.* was susceptible to deltamethrin and DDT in Kabulongo, Katete and Mufweshya, but had low level resistance to deltamethrin (95.6%) and DDT (98%) in Kafue. Resistance to DDT was (3.8%) and deltamethrin (95.2%) was detected in *An. gambiae s.s.* from Kizingezinge.

Similarly in 2010 nine new sites that had only received ITNs as interventions were surveyed for insecticide resistance. In *An. gambiae s.s.* resistance to deltamethrin ranged from 41.8–100% in these sites with resistance to lambda-cyhalothrin being detected in Nyamankalo (83.3%) and DDT in Myooye (69%). Similarly a range of resistance was detected to deltamethrin in *An. funestus s.s.* (66.6–100%), permethrin resistance was also detected in Nanga Farms (90.9%) and to DDT in Myooye (94%) and Nyamankalo (88%) (Table 3 &4).

### Molecular forms and *kdr* mutations

In four localities with pyrethroid and DDT resistant *An. gambiae* and one (Chipepo) with high pyrethroid resistance, PCR assays, to check for the presence of east (leu-ser) (Ranson et al. 2000) and west (leu-phe) (Martinez-Torres et al. 1998) *kdr* mutations were carried out on a random sample. All 159 *An. gambiae s.s* were identified as the molecular S-form and all were negative for east *kdr*. In four of the sites, Chipepo, Chipulkusu, Kizhingezhinge and Mushili all samples carried the west *kdr* mutation. In Myooye, only one sample was homozygous for west *kdr* (Table 5).

**Table 5:** Detection of Leu-Phe (West) *kdr* in survivors populations of *An. gambiae s.s.* tested for DDT and pyrethroid resistance.

Location	Homozygous Leu-Phe mutation	Heterozygous Leu-Phe mutation	Homozygous wild type
**ITN only sites**			
Chipepo	21	0	0
Myooye	1	0	10
**IRS sites**			
Chipulukusu	45	1	0
Kizhingezhinge	20	0	0
Mushili	58	3	0

## Discussion

The Roll Back Malaria, United Nations Millennium Development Goals and World Health Assembly universal access and coverage targets for malaria prevention and treatment have been established to stimulate the reduction in disease transmission. To meet these targets malaria control interventions are now being scaled up [25,26]. In order to obtain and maintain these goals there is a need for continuous surveillance, monitoring and evaluation of malaria control programmes to make informed decisions and guide control efforts.

In Zambia, the initial re-deployment of vector control interventions, ITN (1999) and IRS (2000), was based on minimal empirical evidence. Subsequent monitoring and evaluation has allowed more informed decisions to be made on targeting these interventions. Zambia has achieved high coverage of IRS (20% of households) and ITNs (60% plus of households) as of 2008 [9], and now needs to sustain these efforts.

Prevalence of parasites in children has been frequently used as a surrogate measure for malaria transmission intensity [27]. Previous studies conducted in Zambia, prior to or early into the re-introduction of interventions, as population based surveys, or from hospital based routine surveillance show widely heterogeneous results [7,28,29]. In this study, the overall average *P. falciparum* prevalence in children between the ages of 1 and 14 years was below 10% implying low transmission. This low prevalence has been achieved in part due to effective vector control interventions. Between 2003 and 2008 approximately 5.9 million ITNs (since 2007 all have been LLINs) have been distributed and IRS expanded from 5 to 36 districts, increasing the number of structures sprayed from less than 100,000 to over 1 million [9]. At the same time Zambia expanded clinical control of malaria through improved diagnostics (microscopy and RDTs), introducing ACTs into all clinics and expanding the network of community health workers.

Species density and infectivity measures are important indicators of malaria transmission [20,30]. The endophilic nature of *An. funestus s.s.* and *An. gambiae s.s* makes these species susceptible to both IRS and ITNs, which reduce abundance and sporozoite rates [20,31]. *Anopheles arabiensis* on the other hand is generally exophilic and may be the cause of underlying transmission in Zambia, a topic that warrants further investigation. In this study, both IRS and ITNs are associated with a lower abundance of *An. gambiae* s.s, and *An. funestus s.s.* than *An. arabiensis*. However, IRS is associated with lower abundance than ITNs, with no *An. gambiae s.s* collected from window traps in IRS areas. This may account for the greater impact of IRS on prevalence. Similar results of IRS having a more prompt and powerful impact than ITNs on species abundance has been observed [32,33]. The apparent elimination of *An. gambiae* s.s in IRS areas and suppression of *An. funestus s.s.* and *An. arabiensis* to minimal levels, coupled with the absence of vector infectivity in both IRS and ITNs settings is striking. This effect of reducing abundance and infectivity of malaria vectors results in community wide protection [34,35].

In contrast to the current study, where no infected mosquitoes were collected, previous studies before and early into vector control in Zambia found *P. falciparum* sporozoites in all three major vectors (*An. gambaie* s.s., *An. arabiensis* and *An. funestus s.s.*) [36–38], although this may in part be a function of the different collection methods employed. The identification of *An. nili* and *An. funestus*-*like* species in Zambia, as well as the presence of *An. rivulorum* increases our knowledge of the range of these species. Further work to assess their transmission potential in Zambia is necessary. Indoor interventions have been shown to significantly reduce transmission in neighboring Tanzania. However, a residual transmission of disease remained through outdoor biting of *An. gambiae s.l.* [39]. This may account in part for the transmission that is still observed here, which will not have been detected in the methods used. This requires further investigation if the control programme is to reduce prevalence further.

The evidence of pyrethroid resistance spreading in Africa is mounting [20,40,41] and in some cases has resulted in policy changes in vector control interventions [20,41,42]. High levels of pyrethroid and DDT resistance were detected for the first time in both the *An. gambiae s.s* and *An. funestus s.s* in Zambia during this study, and these findings were subsequently confirmed by other groups. There was significant variation in the frequency of resistance detected between IRS and ITN localities, with higher levels of resistance (P<0.0001) being detected in IRS areas compared to ITNs areas.

Pyrethroid-DDT cross resistance from a common kdr mechanism, has been reported in *An. gambiae s.s* in Africa [21,40,43]. Samples of *An. gambiae s.s* that were pyrethroid and DDT resistant were tested for east and west *kdr* mutations. The west *kdr* mutation was found in all sites tested. This is the most southerly documented detection of this mutation. It is not yet clear whether this resistance has arisen de novo in Zambia or whether it has spread from other locations in Africa, but analysis of introns within the kdr gene would clarify the origin of the resistance. *Anopheles funestus* was also resistant to pyrethroids and DDT at a high frequency. To date there have been no reports of sodium channel mutations in *An. funestus.* The DDT and pyrethroid resistance detected here could arise from two separate metabolic resistance mechanisms, GST and P450 [44] respectively, or may be the first instance of kdr-type resistance in this species documented. Sequencing of the relevant areas of the sodium channel gene in resistant specimens of *An. funestus* is needed to clarify this. However it is notable that the pyrethroid resistant *An. funestus* found in Mozambique is not accompanied by resistance to DDT [41,45]. The detection of multiple resistance in both major vectors of malaria in Zambia may have grave implications for the malaria control programme. It may compromise the efficacy of interventions and potentially lead to the failure of IRS and ITNs based vector control. While this data serves to highlight the threats more information is required on all the underlying mechanisms of insecticide resistance so that a suitable insecticide resistance management plan may be put in place to sustain vector control.

Monitoring malaria cases in Kwa-Zulu Natal, South Africa, picked up the failure of pyrethroids in the IRS programme in the 1990s due to P450 mediated pyrethroid-resistance selection in *An. funestus,* resulting in DDT being successfully reintroduced [46], The resulting decline in malaria was reinforced by a change in drug treatment policy [47]. In Bioko Island, Equatorial Guinea, IRS with pyrethroids had a significant impact on the abundance of *An. funestus* but not on *An. gambiae*. However, a reduced sporozoite level in *An. gambiae* was observed that will have lowered transmission. With the detection of high frequencies of *kdr* in *An. gambiae* on the island a policy change to carbamate was made which appeared to significantly reduce the abundance of *An. gambiae* and maintained the low levels of *An. funestus* [20].

In 2008 the percentage of children aged 1–5 years positive for parasites fell from 25% to <15% in Zambia due to increased malaria control efforts (Chizema-Kawesha, 2010).The monitoring, evaluation and surveillance carried out over a three year period demonstrates the success of Zambia's control programme in reducing the burden of malaria further as signified by reduced prevalence of malarial infection to 6.8%. However, the recent detection of high insecticide resistance to pyrethroids and DDT in *An. gambiae* and *An. funestus* along with *kdr* detection in *An. gambiae* threatens the sustainability of this programme. While the lack of sporozoite positive mosquitoes in the window traps is consistent with reduced levels of transmission the rebound in prevalence of *P. falciparum* in IRS areas may be due to the increased levels of insecticide resistance and the beginning of control programme failure.
